# Humans and Hoofed Livestock Are the Main Sources of Fecal Contamination of Rivers Used for Crop Irrigation: A Microbial Source Tracking Approach

**DOI:** 10.3389/fmicb.2022.768527

**Published:** 2022-06-30

**Authors:** Constanza Díaz-Gavidia, Carla Barría, Daniel L. Weller, Marilia Salgado-Caxito, Erika M. Estrada, Aníbal Araya, Leonardo Vera, Woutrina Smith, Minji Kim, Andrea I. Moreno-Switt, Jorge Olivares-Pacheco, Aiko D. Adell

**Affiliations:** ^1^Escuela de Medicina Veterinaria, Facultad de Ciencias de la Vida, Universidad Andres Bello, Santiago, Chile; ^2^Millennium Initiative for Collaborative Research on Bacterial Resistance (MICROB-R), Santiago, Chile; ^3^Department of Environmental and Forest Biology, College of Environmental Science and Forestry, State University of New York, Syracuse, NY, United States; ^4^Department of Biostatistics and Computational Biology, University of Rochester Medical Center, Rochester, NY, United States; ^5^Escuela de Medicina Veterinaria, Facultad de Agronomía e Ingeniería Forestal, Facultad de Ciencias Biológicas y Facultad de Medicina, Pontificia Universidad Católica de Chile, Santiago, Chile; ^6^Department of Food Science and Technology, Eastern Shore Agricultural Research and Extension Center, Virginia Tech, Painter, Virginia; ^7^Grupo de Resistencia Antimicrobiana en Bacterias Patógenas y Ambientales (GRABPA), Instituto de Biología, Pontificia Universidad Católica de Valparaíso, Valparaiso, Chile; ^8^Escuela Ingeniería Ambiental, Facultad de Ciencias de la Vida, Universidad Andres Bello, Santiago, Chile; ^9^One Health Institute, School of Veterinary Medicine, University of California, Davis, Davis, CA, United States; ^10^Department of Civil and Environmental Engineering, University of California, Davis, Davis, CA, United States

**Keywords:** microbial source tracking, water quality, waterborne pathogens, *Cryptosporidium*, *Giardia*, fecal coliforms, antimicrobial resistance

## Abstract

Freshwater bodies receive waste, feces, and fecal microorganisms from agricultural, urban, and natural activities. In this study, the probable sources of fecal contamination were determined. Also, antibiotic resistant bacteria (ARB) were detected in the two main rivers of central Chile. Surface water samples were collected from 12 sampling sites in the Maipo (*n* = 8) and Maule Rivers (*n* = 4) every 3 months, from August 2017 until April 2019. To determine the fecal contamination level, fecal coliforms were quantified using the most probable number (MPN) method and the source of fecal contamination was determined by Microbial Source Tracking (MST) using the *Cryptosporidium* and *Giardia* genotyping method. Separately, to determine if antimicrobial resistance bacteria (AMB) were present in the rivers, *Escherichia coli* and environmental bacteria were isolated, and the antibiotic susceptibility profile was determined. Fecal coliform levels in the Maule and Maipo Rivers ranged between 1 and 130 MPN/100-ml, and 2 and 30,000 MPN/100-ml, respectively. Based on the MST results using *Cryptosporidium* and *Giardia* host-specific species, human, cattle, birds, and/or dogs hosts were the probable sources of fecal contamination in both rivers, with human and cattle host-specific species being more frequently detected. Conditional tree analysis indicated that coliform levels were significantly associated with the river system (Maipo versus Maule), land use, and season. Fecal coliform levels were significantly (*p* < 0.006) higher at urban and agricultural sites than at sites immediately downstream of treatment centers, livestock areas, or natural areas. Three out of eight (37.5%) *E. coli* isolates presented a multidrug-resistance (MDR) phenotype. Similarly, 6.6% (117/1768) and 5.1% (44/863) of environmental isolates, in Maipo and Maule River showed and MDR phenotype. Efforts to reduce fecal discharge into these rivers should thus focus on agriculture and urban land uses as these areas were contributing the most and more frequently to fecal contamination into the rivers, while human and cattle fecal discharges were identified as the most likely source of this fecal contamination by the MST approach. This information can be used to design better mitigation strategies, thereby reducing the burden of waterborne diseases and AMR in Central Chile.

## Introduction

Fecal pollution in water is associated with negative health impacts, economic losses (including increased health costs), and pathogen contamination of raw foods (Boelee et al., 2019). The World Health Organization reports that diarrheal disease is the second leading cause of death in children under 5-year old, with approximately 1.7 billion cases of childhood diarrheal disease and 525,000 deaths of children per year (WHO, World Health Organization, 2017a). The diarrheal disease mostly results from contaminated food and water sources (WHO, World Health Organization, 2017b). This is worrisome considering that 780 million individuals worldwide lack access to improved drinking-water and 2.5 billion lack improved sanitation (WHO and UNCF, World Health Organization and United Nations Children’s Fund, 2017).

The quality of many water sources has been greatly affected by fecal pathogens from anthropogenic activity such as urban and animal protein production (Pandey et al., 2014). In general, fecal contamination of surface water could be mainly attributed to human activities, such as poor management of fecal waste on farms leading to contaminated runoff into water (Fenton et al., 2021); the excessive use of fertilizers in agriculture (Khan et al., 2018), and the discharge of water treatment plants, since many microorganisms can colonize infrastructure and resist treatment (Newton and McClary, 2019). Some studies have investigated the effect of human activities on diverse pathogen distribution through the water; for example, in a 19-year meta-analysis review, the distribution of some parasites such as *Cryptosporidium* spp. and *Giardia* spp. can be enhanced by climate conditions, water availability, increasing illegal usage of raw wastewater, night soil, and manure amendment for crop soils (Javanmard et al., 2020). For example, the authors indicated that the transmission of parasites is probably limited to rural areas in industrialized districts or those cities where tourism is the main business, while in those regions where traditional animal husbandry is the main business, rain increases the surface water that carries (oo)cysts of parasites dispersed by herds to downstream farms traditionally irrigated with surface waters (Javanmard et al., 2020). Lewis et al. (2005) observed high levels of inputs of fecal coliforms (over 10^8^ fecal coliforms/hectare) in discharges from areas with large cattle herds during precipitation events.

Similarly, Wilkes et al. (2011) reported that the likelihood of detecting *E. coli* O157:H7 was associated with upstream livestock pasture density, with 20% of the detections located where cattle had access to the waterway. Land use is a classification that provides information on the type and intensity of human activities occurring in a given geographical area (LaGro, 2005). Among the land-use classification systems, the most general classification and commonly used for regions and other large-scale applications consist of broad land use categories, such as “agriculture” or “urban and built-up” land use (LaGro, 2005). Therefore, different land uses drive different amounts of fecal material to rivers depending on factors such as human activities, livestock activities, and agricultural activities (Wen et al., 2017; Camara et al., 2019; Weller et al., 2020a) either by direct contact or in indirect ways such as dragged by rain (Ding et al., 2015).

In addition, several studies have reported antimicrobial-resistant *Enterobacteriaceae* in surface water (van Hoek et al., 2015; Guyomard-Rabenirina et al., 2017; Azuma and Hayashi, 2021). Therefore, it is important to consider that bacteria that reach water sources through feces may be carriers of antibiotic resistance genes (ARGs), abetting the spread of antimicrobial resistance (AMR; Pilmis et al., 2020). For instance, multi-drug resistance (MDR) *Salmonella Enteritidis* and Typhimurium, were detected in rivers and irrigation canals, with the higher detection rates observed in rural areas compared to urban and peri-urban areas (Martínez et al., 2017); therefore, land use is an important factor to be considered when evaluating the health risk to human that is in contact with surface water sources. These fecal and antibiotic resistance bacteria (ARB) discharge adversely affect water quality in rivers which is highly relevant to human health, quality of water used for crop irrigation, recreational activities, animal health (both livestock and wildlife) and the environment (Sbodio et al., 2013).

Many of the programs monitoring water quality only focus on measuring Fecal Indicator Bacteria (FIB; Holcomb and Stewart, 2020). These FIB are used because they are commonly found in human and animal feces and are known commensal organisms. Therefore, their presence in water may indicate the presence of pathogens (Korajkic et al., 2018). Among the FIB, the coliform group, consisting of the bacteria *Enterobacter*, *Klebsiella*, *E. coli*, and *Citrobacter*, are used as indicators of fecal pollution of surface water worldwide (Holcomb and Stewart, 2020; Goshu et al., 2021). The presence of high levels of fecal coliforms is a frequently used indicator of the presence of fecally-associated bacteria, including enteric pathogens. These fecal pathogens, specifically protozoa and bacteria, can be transmitted and dispersed in many aquatic environments such as oceans, rivers, creeks, and lakes (Lusk et al., 2017; Devane et al., 2018; Shapiro et al., 2018). Despite this, the major limitation is that they do not provide information regarding the fecal source of contamination as any animal, including humans, can excrete this FIB through their feces (Field and Samadpour, 2007). Research related to determining the level of microbial contamination caused by inland surface waters has not included fecal source contamination. To overcome this limitation of the FIB, Microbial Source Tracking (MST), an approach to determine the contributions of specific hosts of fecal contamination to water sources, has been increasingly used (Raza et al., 2021). Among the MST tools reported in the scientific literature are the protozoan parasites *Cryptosporidium* spp. and *Giardia* spp., as both provide knowledge of host specificity (Ryu et al., 2011; Prystajecky et al., 2014; Xiao et al., 2018). For instance, the genus *Cryptosporidium* is composed of genotypes that are host specific and genotypes that infect various hosts (Xiao et al., 2000), a situation that also occurs with *Giardia* genotypes and assemblages (Feng and Xiao, 2011). *Giardia* is divided into six species based on morphological characteristics, among which *Giardia duodenalis* (synonyms *Giardia lamblia* and *Giardia intestinalis*) infects a variety of mammals, including humans (Cacciò et al., 2005) and will be referred to as *G. duodenalis* throughout this manuscript. Besides, *G. duodenales* have genetic groupings (henceforth assemblages) of *G. duodenalis* and species, each with specific hosts. Therefore, identifying the genotype of *Cryptosporidium* spp. and assemblage for *Giardia duodenalis* can provide information on the host source (animal or human) of origin of the fecal contamination and its zoonotic potential or if they are infective to productive animals such as cows.

Chile provides an ideal condition to study the water quality of rivers because of the geography it presents. In addition, it is a narrow country, allowing an entire river that crossed different land use zones to be sampled on the same day. Water quality is particularly important for Chile as the rivers are the main source of irrigation and drinking water source. Therefore, the objective of this study was to determine the fecal contamination levels using fecal coliforms as indicators, identify the genotypes/assemblages of *Cryptosporidium* and *Giardia*, respectively, to determine the probable source of fecal contamination and detect the presence of antibiotic-resistant bacteria (ARB) in two main rivers of central Chile based on land use (urban, agriculture, livestock, and natural). Finally, we evaluate the association between the presence of fecal coliforms, *Cryptosporidium* spp., *Giardia duodenalis*, and ARB with environmental factors. This study provides information regarding the land uses and sources that contribute the most to rivers’ biological contamination with fecal material and ARB, consequently providing information to create strategies to manage and reduce loads of this fecal contamination to the rivers.

## Materials and Methods

### Study Sites

Sampling sites corresponded to sites representing different land-use areas in the Maipo and Mapocho Rivers, both located in the central macrozone of Chile. The Maipo crosses the Metropolitan and Valparaiso Regions. The Maule crosses the Maule Region of the country. The Maipo is the primary source of drinking water for the Metropolitan region and supplies approximately 70% of the drinking water and 90% of the irrigation demands (DGA, Dirección General de Aguas, 2004a). Similarly, the Maule provides irrigation to approximately 118,263 hectares and drinking water to approximately 99,6% of the urban population (DGA, Dirección General de Aguas, 2004b). Fecal coliforms levels in the Maipo and Maule Rivers have exceeded levels permitted by the Chilean water quality standard (INN, Instituto Nacional de Normalización, 1978). For example, fecal coliforms in the Maipo River ranged between <2 and 8.50 × 10^6^ fecal coliforms/100 ml in past reports (DGA, Dirección General de Aguas, 2004a), while the Maule River between 540 and 1.1 × 10^4^ fecal coliforms/100 ml (DGA, Dirección General de Aguas, 2004b).

Land use throughout each river system was categorized using information from the Land Use Atlas of the Chilean Military Geographic Institute (Instituto Geográfico Militar de Chile, 2005). The land-use categories considered were as: (i) natural, (ii) agriculture, (iii) urban, and (iv) livestock and/or forestry. The Maipo and Maule Rivers share similar land use classifications, allowing a comparison between land use zones and both river courses. The natural zone represents an area with low influence of urban, agricultural, and/or livestock activity. At the same time, the other sites have large urban, agricultural, or livestock/forestry activities, respectively. Both the Maipo River’s natural and agricultural land use areas receive the discharge of two wastewater treatment plants (WWTP), causing these zones to receive urban discharges.

**Figure 1:**
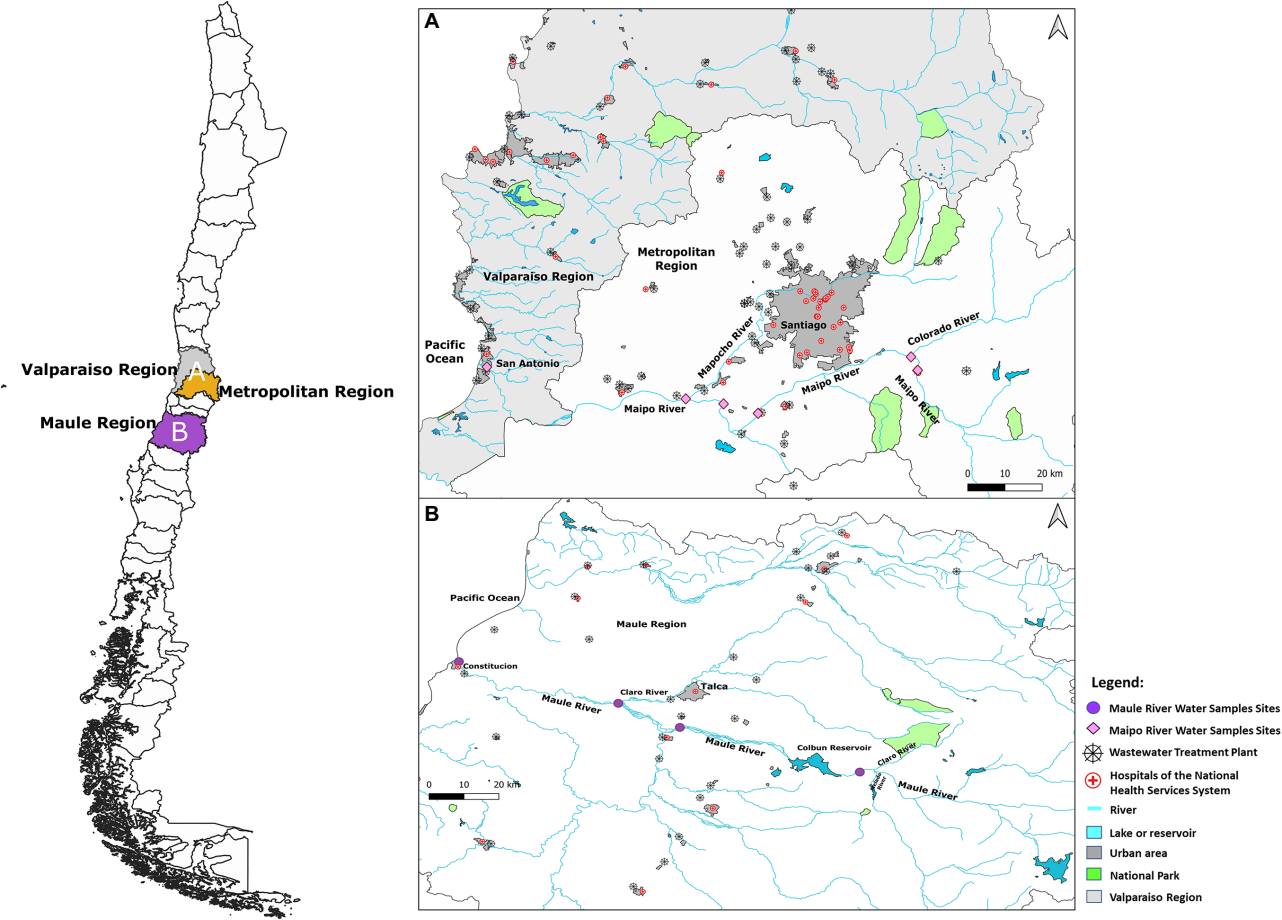
Sampling sites located in: **(A)** the Maipo River system which crosses the Metropolitan and Valparaiso Regions (pink diamonds); and **(B)** the Maule River, which crosses the Maule region (purple circles). Map were created using Quantum GIS version (QGIS) 3.18-Zürich open-source software (qgis.org/es/site/) under a Creative Commons license (www.gny.org/licenses).

For this reason, river water samples were collected before and after WWTP effluent discharge to determine whether this action influences the microbial source tracking by introducing *Cryptosporidium* spp. and *Giardia* spp. species that are human host specific into agricultural or natural areas. The selected urban sites corresponded to the discharge of rivers that have an important influence of major cities; for instance, the Mapocho River crosses the Chilean capital and major metropolis (Santiago) and discharges in the studied site in the Maipo River, and the Claro River receives the urban influence of the capital and major city in the Maule region (Talca) and discharges in the area sampled in the Maule River (Figure 1).

### Water Samples

Water samples were collected from each river every 3 months between June 2017 and April 2019. The timing of sampling was selected to ensure sampling during each season: summer (December–March), fall (March–June), winter (June–September), and spring (September–December). Fall and winter sampling events correspond to the rain event, while spring and summer sampling events correspond to the dry season. All sampling sites were georeferenced using Google Maps. Water was collected in a sterile plastic bottle at each sampling site, which was rinsed three times in the river before collecting the sample. The sample was collected by submerging the bottle at least 10 cm below the surface. Immediately after sample collection, bottles were placed in a cooler and transported on ice. Samples were all processed within 24 h. Physical parameters of pH, water temperature (°C), conductivity (μS or mS), salinity (ppm or ppt), and total dissolved solvents (ppm or ppt) were measured *in-situ* in each site using the Yalitech AM 006 Waterproof Multiparameter Meter Combo 6. Weather conditions (sunny, cloudy, or raining) were also recorded at the time of sampling.

### Fecal Coliforms Quantification

Water samples (15 ml) were used for fecal coliform enumeration following the Chilean Regulation NCh2313/22:1995 protocol for the detection of fecal coliforms in wastewater using EC medium [Instituto Nacional de Normalización (INN), 1995] to determine if each sample was complaint with Chilean water quality standards (INN, Instituto Nacional de Normalización, 1978) and whether the river complied with the water quality standards for surface waters. Specifically, based on this regulation, we considered that a sample was compliant with the standard if fecal coliforms levels were below 1,000 fecal coliforms /100 ml of water; compliance is based on the assumption that fecal coliform levels above 1,000 fecal coliforms/100 ml is indicative of fecal contamination. The most probable number (MPN) of fecal coliforms in 100 ml of water was conducted following the instructions of the Chilean Regulation NCh2313/22:1995 protocol.

### Protozoa Quantification and Sequencing

To identify possible sources of fecal contamination, 20 L water samples were concentrated to 200 ml using Hollow-Fiber Ultrafiltration and B Braun DIACAP HIPS 20 (catalog code: 10-10-149144-5) hemodialysis filters following the methodology described by Hill et al. (2005) and Bambic et al. (2011). *Cryptosporidium* spp. oocysts and *Giardia duodenalis* cysts in the ultra-filtrated water were quantified according to the United States Environmental Protection Agency (EPA) method 1623 (Miller et al., 2005). *Cryptosporidium* spp. oocysts and *Giardia duodenalis* cysts were concentrated using Dynabeads *Cryptosporidium*/*Giardia* Combo kit (Applied Biosystems, IDEXX Laboratories Inc., Maine, United States) immunomagnetic separation (IMS). IMS was conducted following Miller et al. (2005) with some modifications. Since metallic sediments interfered with the IMS step, a sediment wash was performed before adding the 100 μl IMS Dynal *Cryptosporidium* spp. and *Giardia duodenalis* beads to remove metallic sediments. Levels of each target were stained using Waterborne kit reagents (Aqua-Glo G/C Direct; Waterborne Inc., New Orleans, LA) per the manufacturer’s instructions and enumerated according to Environmental Protection Agency (EPA) method 1623 (Miller et al., 2005). Briefly, 3-well slides (well diameter 14 mm) were stained with fluorescein isothiocyanate (FITC), and DAPI (4,6-diamidino-2-phenylindole) and the entire well was examined for protozoa using an Olympus epifluorescence microscope. Organisms were visualized at ×200 magnification, and identifications were confirmed at × 400 (Miller et al., 2005). *Cryptosporidium parvum* and *C. hominis* oocysts were identifiable as 5–7 μm-diameter spheres with apple green outlines, often with a midline seam. *Cryptosporidium* spp. oocysts were identified as 5–7 μm-diameter spheres outlined in apple green and often with a midline seam, whereas *Cryptosporidium andersoni/C. muris* like organisms were identified as 5-by-7-μm elliptical forms (Miller et al., 2005). *Giardia* cysts were also apple green but oval and 9–14 μm long (Miller et al., 2005). The same experienced microscopist read all slides. As described below, samples were then subjected to DNA extraction and conventional PCR for confirmation and sequencing of *Cryptosporidium* spp. and *Giardia duodenalis*.

### Protozoa Molecular Analyses

A modified version of a previously published method (Miller et al., 2005) was used for extracting DNA from all the slides. After protozoa quantification the slide well for each sample was scraped with a scalpel blade and washed with sterile PBS into a separate microcentrifuge tube. Proteinase K (40 μl) was added to the sample and kept at 56°C overnight for sample digestion. DNA was extracted using Qiagen DNA Minikit (Qiagen Inc., Valencia, CA), and subsequently purified using a QIAamp column. Extracted DNA was stored at −20°C until PCR analysis.

The *Cryptosporidium* PCR was based on molecular characterization of the 18S rRNA gene using nested primers that amplify ~825 bp product (Xiao et al., 1999, 2000), and using conventional primers that amplified a 298-bp DNA fragment (Morgan et al., 1997). Amplification conditions were described by Adell et al. (2014). The *Giardia* PCR was based on molecular characterization of the glutamate dehydrogenase (GDH) and the SSU rRNA genes. GDH confirmation amplifies a ~432-bp fragment using a semi-nested-PCR protocol and primers (Read et al., 2004), while a ~292 bp final fragment of the SSU rRNA gene was amplified using a nested PCR protocol and primers (Appelbee et al., 2003). All primers are listed in Supplementary Table S1. All DNA was purified and submitted to Macrogen (Macrogen, Inc. Korea) for sequencing.

The protozoan parasites *Cryptosporidium* spp. and *Giardia* spp. have been used as an MST tool because both provide knowledge of host specificity (Ryu et al., 2011; Prystajecky et al., 2014; Xiao et al., 2018). For instance, the genus *Cryptosporidium* is composed of genotypes that are host specific and genotypes that infect various hosts (Xiao et al., 2000). For example, the major hosts for *C. parvum* are cattle, other ruminants, and humans; for *C. andersoni* cattle and bactrian camels; for *C. baileyi*, poultry and birds; for *C. meleagridis*, humans, turkeys, and birds; for *C. canis*, dogs; and other 10 species that have specific hosts (Xiao et al., 2004; Cacciò et al., 2005).

A situation also occurs with *Giardia* genotypes and assemblages (Feng and Xiao, 2011). *Giardia* spp. are divided into six species based on morphological characteristics, among which *Giardia duodenalis* (synonyms *G. lamblia* and *G. intestinalis*) infects a variety of mammals, including humans and livestock (Cacciò et al., 2005) and will be referred to as *G. duodenalis* throughout the manuscript. *G. duodenalis* also have several genetic groupings (henceforth assemblages), each of them having specific hosts. Thus, assemblage A can infect humans, other primates, livestock, dogs, cats, rodents, and other wild animals; similarly assemblage B is specific to humans and other primates, dogs, some species of wild mammals, assemblage C and D (also reported as *Giardia canis*) are specific to dogs and other canids; assemblage E (also reported as *G. bovis*) to cattle and other hoofed livestock; assemblage F (also reported as *G. cati*) to cats; and assemblage G (or *G. simondi*) to rats (Cacciò et al., 2005; Monis et al., 2009).

Therefore, in our study the idnetifying the genotype of *Cryptosporidium* and assemblage for *Giardia duodenalis* will be used to provide information on the host source (animal or human) of origin of the fecal contamination, its infective potential to productive animals (cattle), and its zoonotic potential.

### Protozoa Phylogenetic Analysis

To allow for the identification of *G. duodenalis* and *Cryptosporidium* subtypes associated with specific hosts and, therefore, fecal sources, three custom databases (*Giardia*_SSU, *Giardia*_GDH, and Crypto_18S) were built by searching the NCBI-nr database and publicly available literature. DNA sequences were aligned using ClustalW-2.1 (GNU Lesser GPL, 2010), and phylogenies were built in Molecular Evolutionary Genetics Analysis (MEGA) version 10.2.5 software (Kumar et al., 2018) using maximum likelihood and Tamura 3. Phylogenetic tree topological reliability was determined using the bootstrap method, with 1,000 replicates and bootstrap lower than 70% were deleted from the tree. Accession numbers with the nucleotide sequence data for PCR products used for phylogenetic classification obtained during this study are available in Supplementary Table S2.

### Statistical Analysis

Conditional inference trees (CTree) are a type of modified classification and regression tree (CART) to address fundamental limitations of traditional CART algorithms (e.g., bias toward using categorical variables with many levels/continuous variables for splits over categorical variables with fewer levels). It is important to note that this is a multivariable and not a multivariate analysis and is a standard approach used in environmental microbiology literature, including by water-focused studies throughout the past decade (Bae et al., 2010; Strawn et al., 2013; Verhougstraete et al., 2015; Weller et al., 2016, 2020a; Green et al., 2021; Guo and Lee, 2021; Hannan and Anmala, 2021). Ctrees and CARTs are ideal tools for capturing complex (e.g., hierarchical) relationships within data and are thus, well suited for observational studies in non-controlled environments (Kuhn and Johnson, 2016). For example, identifying combinations of factors (specific scenarios) associated with an increased or decreased likelihood of detecting each microbial target, which is something regression-based approaches cannot do (Kuhn and Johnson, 2016). Tree-based approaches also create easy-to-understand visuals and do not rely on significance testing, thereby avoiding limitations associated with multiple comparison testing (Kuhn and Johnson, 2016). Moreover, tree-based analyses do not generate effect estimates or odds ratios to quantify the strength of associations, which is worth mentioning as other studies have reported substantial variability in water quality within a waterway over time; therefore, the duration of our study was insufficient to generate reliable effect estimates (Kuhn and Johnson, 2016; Weller et al., 2020b,c).

Conditional tree analysis was used to characterize the relationship between environmental factors and (i) fecal coliform levels log transformed (log10 fecal coliforms/100-ml), (ii) whether fecal coliform levels exceeded the Chilean water quality standard of 1,000 fecal coliforms/100-ml (Yes/No), and (iii) if *Giardia* spp. were detected by PCR (Yes/No); tree analyses were implemented using the mlr and party packages and visualized using the partykit as previously described (Strobl et al., 2007a,b, 2009; Boulesteix et al., 2015; Weller et al., 2020b,c). Briefly, hyperparameters were tuned, models trained, and performance assessed using three-fold cross-validation repeated 10 times; cross-validation was performed to optimize R-squared (for continuous outcomes) and area under the curve (AUC; for binary outcomes). For imbalanced, binary outcomes (i.e., *Giardia* detection versus non-detection), SMOTE resampling was performed as part of tuning (Kuhn and Johnson, 2016; Weller et al., 2021).

To minimize the potential for overfitting we: (i) tuned the maxdepth parameter, (ii) limited the upper bound of maxdepth to 6, and (iii) used a mincriterion of 0.95. Conditional forests analysis was used since it is robust to correlation and missingness in explanatory factors (by enabling surrogate splits), can handle both categorical and continuous outcomes, and can capture complex, hierarchical relationships (e.g., interactions) between explanatory factors. The environmental features considered were the river system (i.e., Maule versus Maipo); predominant land use at the site [i.e., plant-based agriculture (henceforth agricultural), livestock operations, natural, urban sites not at a wastewater discharge (henceforth urban) and urban sites with a wastewater discharge (henceforth treated urban)]; season (i.e., wet versus dry); if feces, garbage, or livestock were present at the sampling site; pH; water temperature; total dissolved solids; salinity; if it rained during the 24 h before sampling; and weather conditions at the time of sampling (i.e., rainy, sunny, partly cloudy, or cloudy). For *Giardia*, coliform levels and if coliform levels met the Chilean water standard (i.e., were above or below 1,000 fecal coliforms/100-ml) were also considered explanatory factors. Separately, nonparametric Spearman’s one-tailed tests were conducted to assess the correlation between continuous outcome variables (i.e., MPN of fecal coliforms/100 ml, *Giardia* cysts/100 ml, and *Cryptosporidium* oocysts/100 ml) to enable comparison of the findings reported here with previous studies (e.g., Wu et al., 2011; Korajkic et al., 2018). A generalized linear model (GLM) was implemented to determine whether compliance with the Chilean water quality standard (i.e., fecal coliforms above versus below 1,000 fecal coliforms/100-ml) was associated with protozoa detection (i.e., if *Cryptosporidium*, *Giardia*, or no protozoa were detected). In both analyses (Spearman’s correlation and GLM), differences with *p* < 0.05 were statistically significant. All statistical analyses were performed using RStudio v1.1.456 or R v3.6.3 (R Core Team, 2020; RStudio Team, 2020).

### Detection of Antimicrobial Resistant *Escherichia coli*

To determine if multi-drug resistant *E. coli* were present in water samples, a subset of samples (from the first four of the eight total samplings) were tested (*n* = 48 total samples were tested). Briefly, 1 ml ultra-filtered samples were enriched in 5 ml of Buffer Peptone Water and incubated at 37°C for 24 h. Following incubation, 100 μl of the samples were streaked onto plates with MacConkey agar supplemented with Ciprofloxacin (1 mg/L), Cefotaxime (1 mg/L), and Tetracycline (4 mg/L) and incubated at 37°C for 24 h. Colonies were selected according to morphology using a magnifying glass. This selection was made according to standard patterns of color (pink), as described previously (Jung and Hoilat, 2020). All isolates were identified using MALDI-TOF (BioMérieux, Marcy l’Etoile, France) and only the isolates identified as *E. coli* were tested against a panel of 15 antibiotics using the disk diffusion method to assess their antimicrobial susceptibility profile following CLSI guidelines (Clinical and Laboratory Standards Institute, 2018a). The antibiotic used were: Ampicillin (AMP, 10 μg); Cefazolin (CFZ, 30 μg); Ceftriaxone (CRO, 30 μg); Cefepime (FEP, 30 μg); Imipenem (IPM, 10 μg); Ciprofloxacin (CIP, 5 μg); Amikacin (AMK, 30 μg); Gentamicin (GEN, 10 μg); Fosfomycin/Trometamol (FOF, 200 μg); Amoxicillin-Clavulanate (AMC, 30 μg); Cefuroxime (CXM, 30 μg); Aztreonam (ATM, 30 μg); Chloramphenicol (CHL, 30 μg); Tetracycline (TET, 30 μg); and Trimethoprim/Sulfamethoxazole (SXT, 1.25/23.75 μg). All antibiotics were supplied by OXOID (Hampshire, England). Isolates resistant to three or more antimicrobial classes were cataloged as MDR (Multi-Drug-Resistant Bacteria) following previously standardized criteria (Magiorakos et al., 2012).

### Detection of Environmental Resistant Bacteria

To further characterize environmentally resistant bacteria, 50 ml of water samples from Maipo and Maule Rivers, were passed through a 0.22-μm filter. This filter was resuspended in 0.85% saline solution, and 200 μl were seeded in TSB and R2A agar media in 120 mm square plates. These plates were incubated at 37°C, 25°C and 15°C, and the colonies were classified according to the standard pattern: shape, color, texture, and shape of the colony border and designated as morphotypes (Higuera-Llantén et al., 2018). A total of 2631 morphotypes were isolated at all sampling points, media, and temperatures. Minimum Inhibitory concentrations (MICs) for Ampicillin (AMP), Erythromycin (ERY), Ceftazidime (CAZ), Imipenem (IMI), Ciprofloxacin (CIP), Gentamicin (GEN) and Tetracycline (TET) were determined by agar dilution methods as recommended by CLSI (Clinical and Laboratory Standards Institute, 2018b). Briefly, serial two-fold dilutions ranging from 0.1 to 2,048 μg/ml of any antibiotic were added into Mueller-Hinton agar (Difco). After overnight growth, all isolates were resuspended in 0.85% saline solution and inoculated in plates using a 96-needle replicator. The isolates that grew at 37°C were incubated for 24 h, while those that grew at 25°C and 15°C were incubated for 48 h. A Mueller-Hinton agar plate without antibiotics was used for growth and possible contamination control. These experiments were performed in triplicate. MIC was defined as the lowest concentration of antibiotic that inhibits the growth completely at least in two of three plates seeded at any temperature. As a reference, we used the strain *Escherichia coli* ATCC 25922, as recommended by CLSI (Clinical and Laboratory Standards Institute, 2018b).

## Results

### Coliform Detection and Evaluation of Their Association to Environmental Factors

Fecal coliform levels in the Maule River ranged between 1 and 130 MPN/100-ml, not exceeding the Chilean water quality standard of 1,000 fecal coliforms/100-ml (Table 1). Conversely, fecal coliform levels in the Maipo ranged between 2 and 30,000 MPN/100-ml in 11 samples exceeding the water quality standard. Most of the Maipo River samples that exceeded the standard were mainly collected from urban sites. In both rivers, fecal coliform levels varied based on season, with higher levels during the dry season (mid-September to mid-March) compared to the wet season (mid-March to mid-September; Table 1). Fecal coliform levels also appeared to vary based on land use (Table 1). The samples with the highest coliform levels were collected from urban sites in both rivers, while the samples with the lowest coliform levels were collected from natural sites (Table 1). While the range in fecal coliforms levels from treated urban sites (i.e., sites in the river at a wastewater discharge; <2–8,000 CFU/100-ml) overlapped the range for urban sites (i.e., sites not at a discharge; 34–30,000 CFU/100-ml), the mean level in treated sites (6,887 CFU/100-ml) was considerably lower than the mean in urban sites (32,705 CFU/100-ml); this difference was even more pronounced when only Maipo River sites were considered.

**Table 1:** Fecal coliform levels (MPN/100 ml) for each sample collected in the Maule and Maipo Rivers between 2017 and 2019.

River	Site [predominant land use(s)]	Fecal coliform counts (MNP/100 ml) by season and year							
		2017		2018				2019	
		Winter	Spring	Summer	Fall	Winter	Spring	Summer	Fall
Maule	Site 1 (natural)	<2	13	<2	13	11	<2	23	2
	Site 2 (agricultural)	4	13	130	50	4	23	80	23
	Site 3 (urban)	50	70	30	30	110	130	17	23
	Site 4 (livestock/forestry)	22	2	30	30	30	17	22	50
Maipo	Site 5 (natural)	17	27	300	80	70	80	170	50
	Site 6 (treated urban)	200	4	30	4	8,000^1^	80	1,700^1^	<2
	Site 7 (natural)	50	27	130	50	230	70	33	80
	Site 8 (agricultural)	220	130	700	230	800	5,000^1^	1,300^1^	130
	Site 9 (treated urban)	80	27	800	130	230	1,100^1^	300	300
	Site 10 (agricultural)	27	800	1,300^1^	800	700	1,100^1^	500	700
	Site 11 (urban)	34	280	30,000^1^	800	1,100^1^	700	13,000^1^	8,000^1^
	Site 12 (livestock)	280	280	130	220	300	220	130	500

1 Fecal coliform level exceeded the maximum limit of 1,000 fecal coliforms/ml established by the Chilean water quality standards (INN, Instituto Nacional de Normalización, 1978).

**Figure 2:**
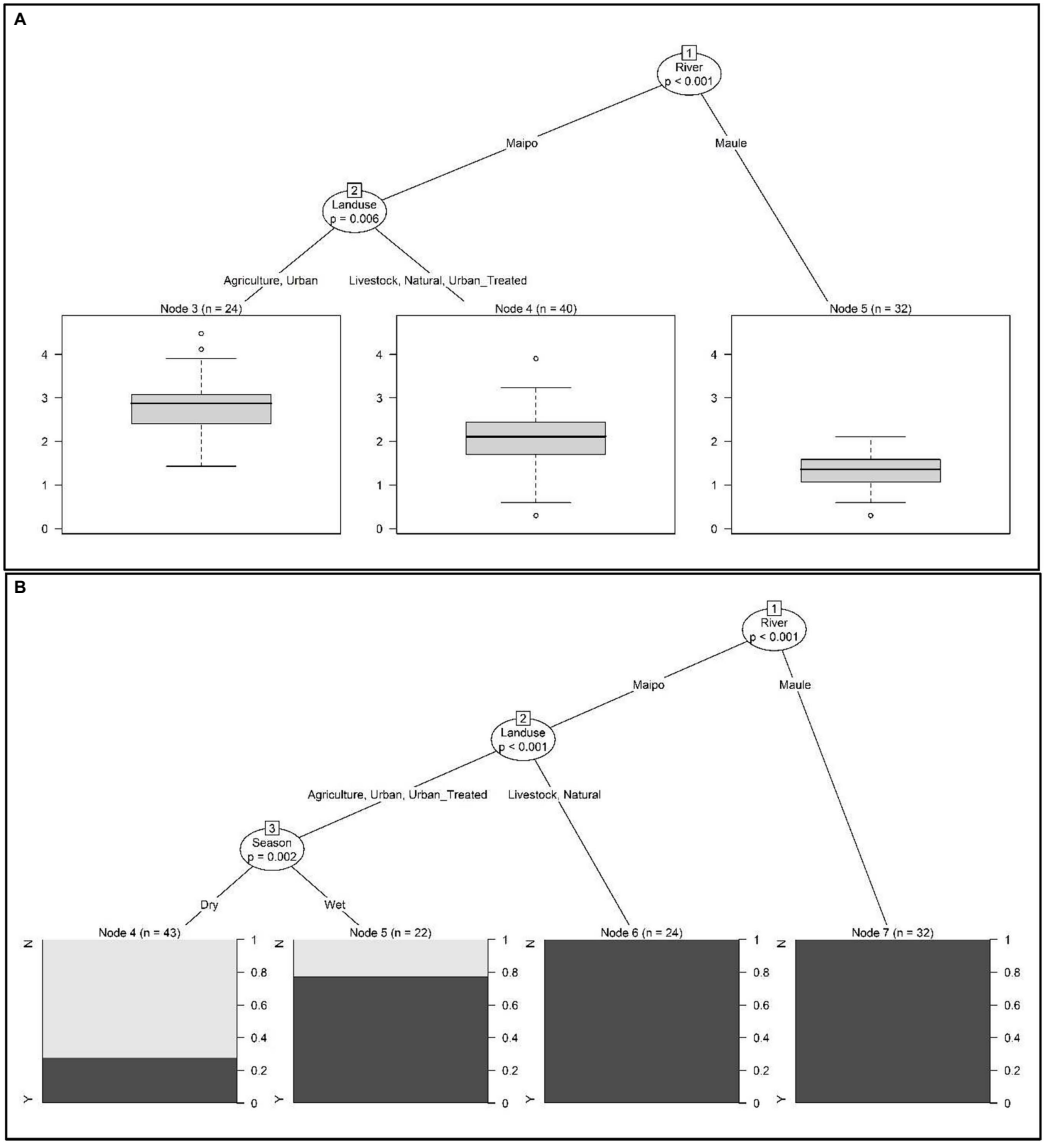
Conditional inference trees were used to visualize hierarchical relationships between environmental factors and **(A)** fecal coliform levels (log10 MPN/100-ml) in water samples, and **(B)** if fecal coliform levels were above or below the Chilean water quality standard (i.e., above or below 1,000 CFU/100-ml). In **(A)** the boxplot shows the distribution of log10 fecal coliform levels in samples that met the given condition. In **(B)** the black bar in each plot shows the probability of the water sample being non-compliant with the Chilean water quality standard when the given conditions were met. For example, fecal coliform levels were highest in Maipo River samples collected from site with predominantly agricultural and urban land uses (that were not at a wastewater discharge site).

According to conditional tree analysis, fecal coliform levels were significantly lower in the Maule compared to the Maipo River (*p* < 0.001; Figures 2A,B). Specifically, fecal coliforms were lowest in Maule River sites and highest in samples from Maipo River sites where the predominant land use was agricultural (i.e., plant-based production) or urban (i.e., not at a wastewater discharge) as opposed to livestock, natural, or treated urban (i.e., urban sites at a wastewater discharge; Figure 2A). All samples collected from the Maule River met the Chilean water quality standard, and this river was significantly associated with if a water sample was compliant with the standard (*p* < 0.01; Figure 2B). For Maipo River, samples were most likely to be non-compliant if collected from sites where the predominant land use was agricultural, urban, or treated urban during the dry season. Samples were most likely to be compliant if collected from sites where the land use was natural or livestock-based production (Figure 2B). For urban, treated urban and agricultural sites, samples were more likely to meet the standard if collected during the wet season (*p* = 0.002; Figure 2B).

### Evaluation of *Cryptosporidium* spp. and *Giardia duodenalis* as Microbial Source Tracking Tools in Maipo and Maule Rivers and Their Associations With Environmental Factors

**Figure 3:**
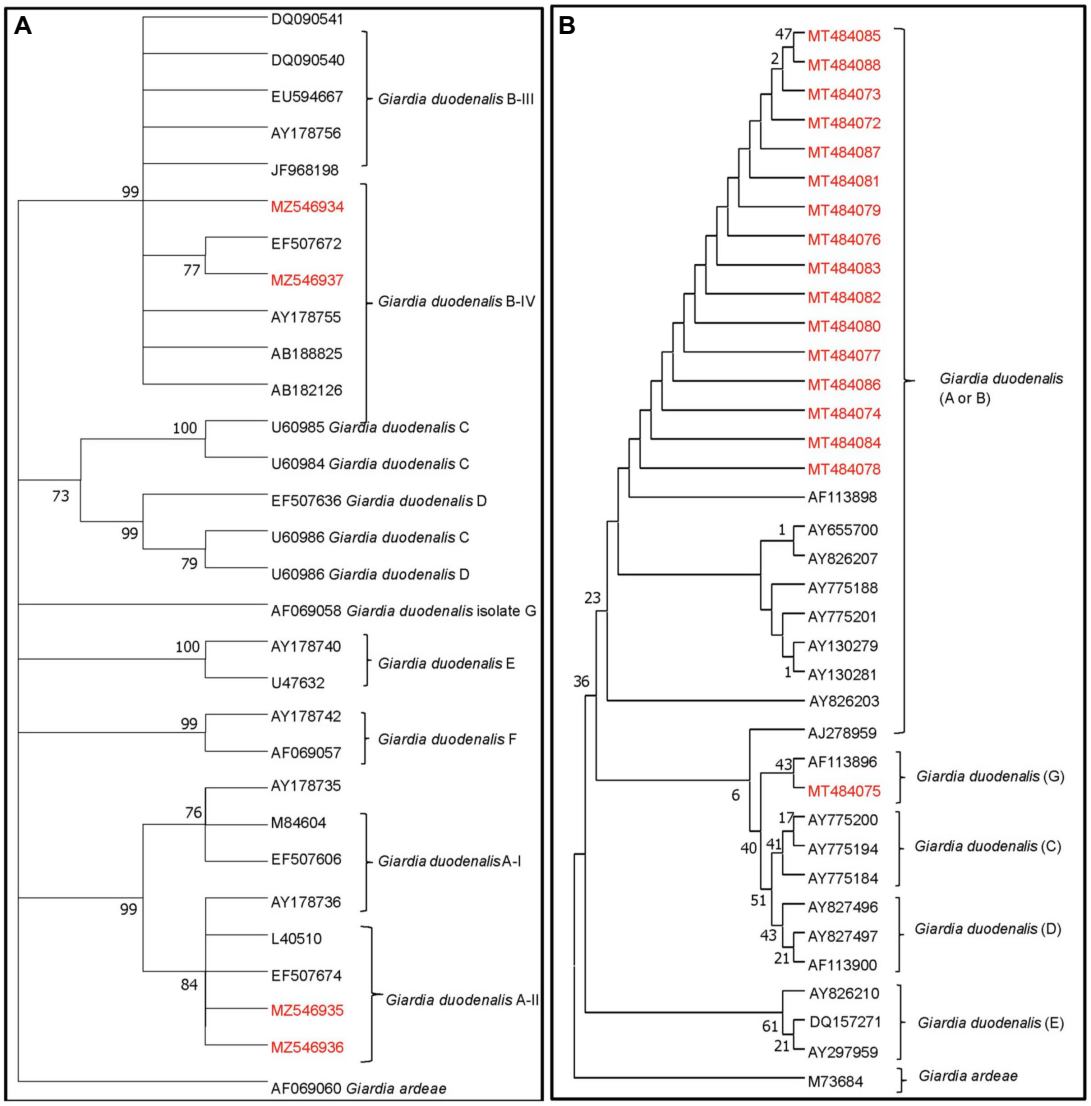
Maximum likelihood phylogenetic analysis of *G. duodenalis* detected from river water samples. The analysis was constructed by using the Tamura 3 parameter model with MEGA 7.0 and the bootstrap values were calculated with 1,000 replicates. The analysis is based on **(A)** the GDH gene and **(B)** ssuRNA. The phylogenetic tree was rooted to *G. ardeae*. Depending on the assemblages of *G. duodenalis* determined by the different clades of the phylogenetic tree, the possible host source of fecal contamination could be inferred. The numbers at branch nodes represent bootstrap values greater than 70. Reference sequences included in the analysis are shown with their respective GenBank accession numbers. *G. duodenalis* strains characterized in this study are shown in red text.

**Figure 4:**
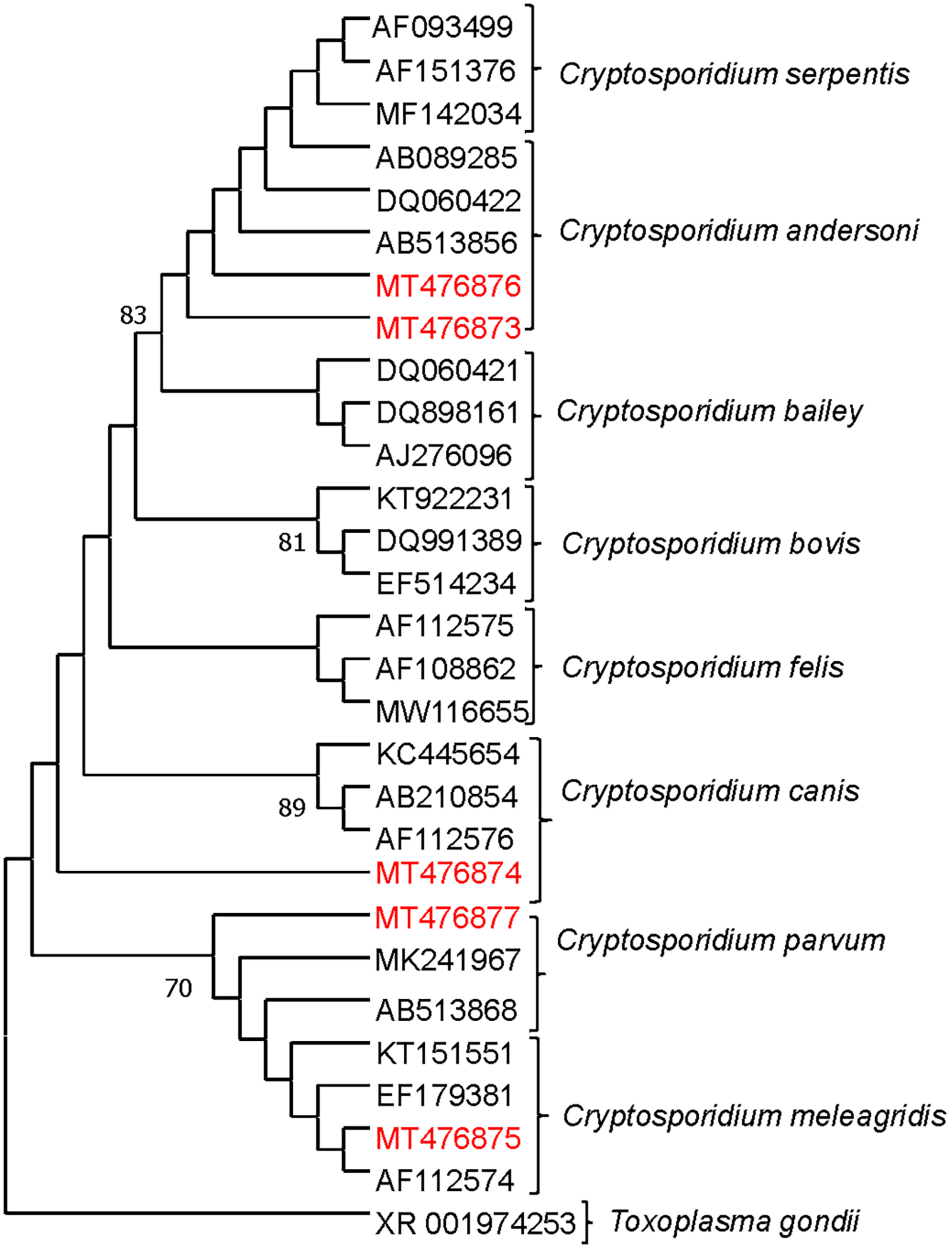
Maximum likelihood phylogenetic analysis of *Cryptosporidium* detected from river water samples. The analysis was constructed by using Tamura 3 parameter model with MEGA 7.0 and the bootstrap values were calculated with 1,000 replicates. The analysis was based on the SSU gene. The phylogenetic tree was rooted to *Toxoplasma gondii*. The numbers at branch nodes represent bootstrap values greater than 70. Reference sequences included in the analysis are shown with their respective GenBank accession numbers. *Cryptosporidium* strains characterized in this study are shown in red text. Based on the *Cryptosporidium* species determined by the different clades of the phylogenetic tree, the possible host source of fecal contamination could be inferred. *Indicates that the sequence was inferred (or confirmed) using blast on the NCBI platform.

Higher *Cryptosporidum* spp. counts were found in water samples collected from the Maipo River (range = 0–60 oocysts/10 L; mean = 3.18 oocysts/10 L) compared to the Maule River (range = 0–31 oocysts/10 L; mean = 2 oocysts/10 L; Supplementary Figure S1). Higher *Giardia* spp. counts were also found in the Maipo (range = 0–171 cysts/10 L, and a mean of 7 cysts/10 L) compared to the Maule River (range = 0–4 cysts/10 L, and a mean of <1 cysts/10 L; Supplementary Figure S1). In Maipo River samples, two assemblages of *G. duodenalis* were identified, including assemblages A or B (Figure 3; Supplementary Tables S2, S3). Only two *G. duodenalis* were detected in water samples collected from the Maule River. One of them corresponds to assembly A or B, and the other is assembly G. Also, four *Cryptosporidium* spp. were identified in the Maipo River, including *C. parvum*, *C. andersoni*, *C. meleagridis*, and *C. canis* (Figure 4; Supplementary Tables S2, S3). Sequencing analysis did not identify any *Cryptosporidium* spp. in the Maule River (Figure 4; Supplementary Tables S2, S3).

**Figure 5:**
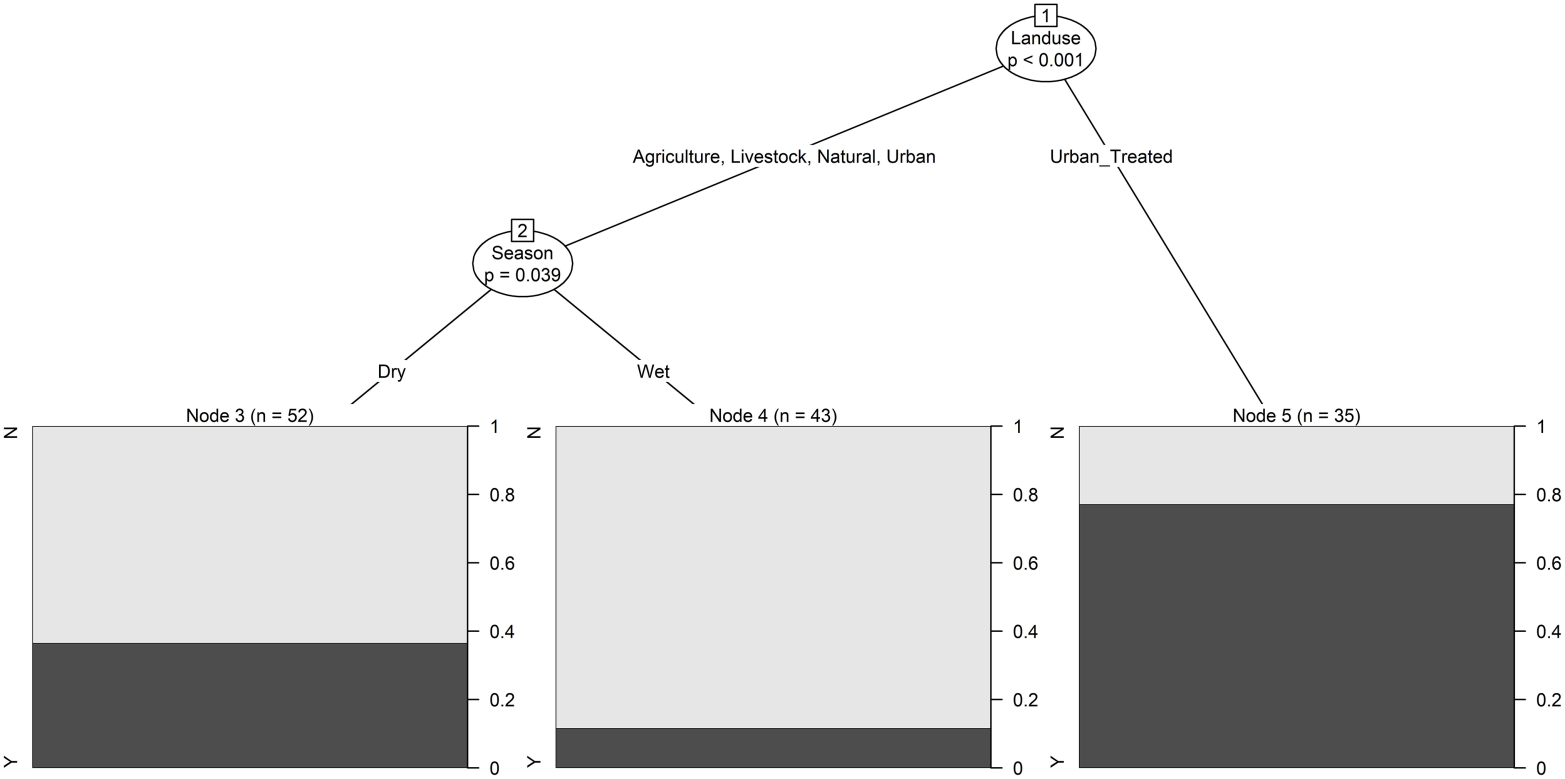
Conditional inference trees were used to visualize hierarchical relationships between environmental factors and if *Giardia* cysts were detected or not. The black bar in each plot shows the probability of *Giardia* being detected when the given conditions were met. For example, the lowest probability of *Giardia* detection was in samples collected from sites with a predominant land use that was agricultural, livestock, natural or urban (as opposed to sites at a wastewater discharge) in the wet season.

Most *Cryptosporidium*-positive samples were collected during the wet season. Whereas *Giardia*-positive samples were collected in both seasons, more positives were collected during the dry season. While fecal coliform levels were highest in samples collected from agricultural and urban sites, *G. duodenalis* assemblages A or B, and *C. meleagridis* were only detected in samples collected from agricultural sites (i.e., sites with plant-based production; Table 1; Figure 3; Supplementary Tables S2, S3). While all, except one sample, collected from sites upstream of wastewater discharges (i.e., natural or agricultural sites) were *Giardia*-negative, multiple samples (*n* = 8 samples) collected downstream of wastewater discharges (i.e., treated urban sites) were positive for *G. duodenalis* assemblages A or B (Supplementary Table S3). Indeed, conditional tree analysis indicated that the likelihood of detecting *Giardia* by PCR was greatest in samples collected downstream of wastewater discharges (i.e., treated urban sites; *p* < 0.001) and lowest in samples collected from all other sites during the wet season (Figure 5).

### Correlation Between Fecal Coliforms and Protozoa Detected in River Water Samples

Spearman’s correlation analysis indicated that coliforms counts (MPN/100 ml) significantly, and positively correlated with *Giardia* counts (cysts/100 ml; correlation coefficient = 0.31; *p* = 0.002) but were not significantly correlated with *Cryptosporidium* counts (oocysts/100 ml; correlation coefficient = 0.03; *p* = 0.75). However, the lack of a correlation between coliform and *Cryptosporidium* spp. levels may be due to the large number of *Cryptosporidium*-negative samples. According to GLM analysis, neither *G. duodenalis* (OR = 0.84; *p* = 0.896; 95% Confidence Interval = 0.20, 6.33) or *Cryptosporidium* spp. (OR = 0.48; *p* = 0.531; 95% Confidence Interval = 0.06, 9.95) detection were associated with fecal coliforms levels being compliant with the Chilean water quality standard of 1,000 CFU of fecal coliforms/100-ml.

### Antimicrobial Resistant *Escherichia coli* and Environmental Resistant Bacteria

**Figure 6:**
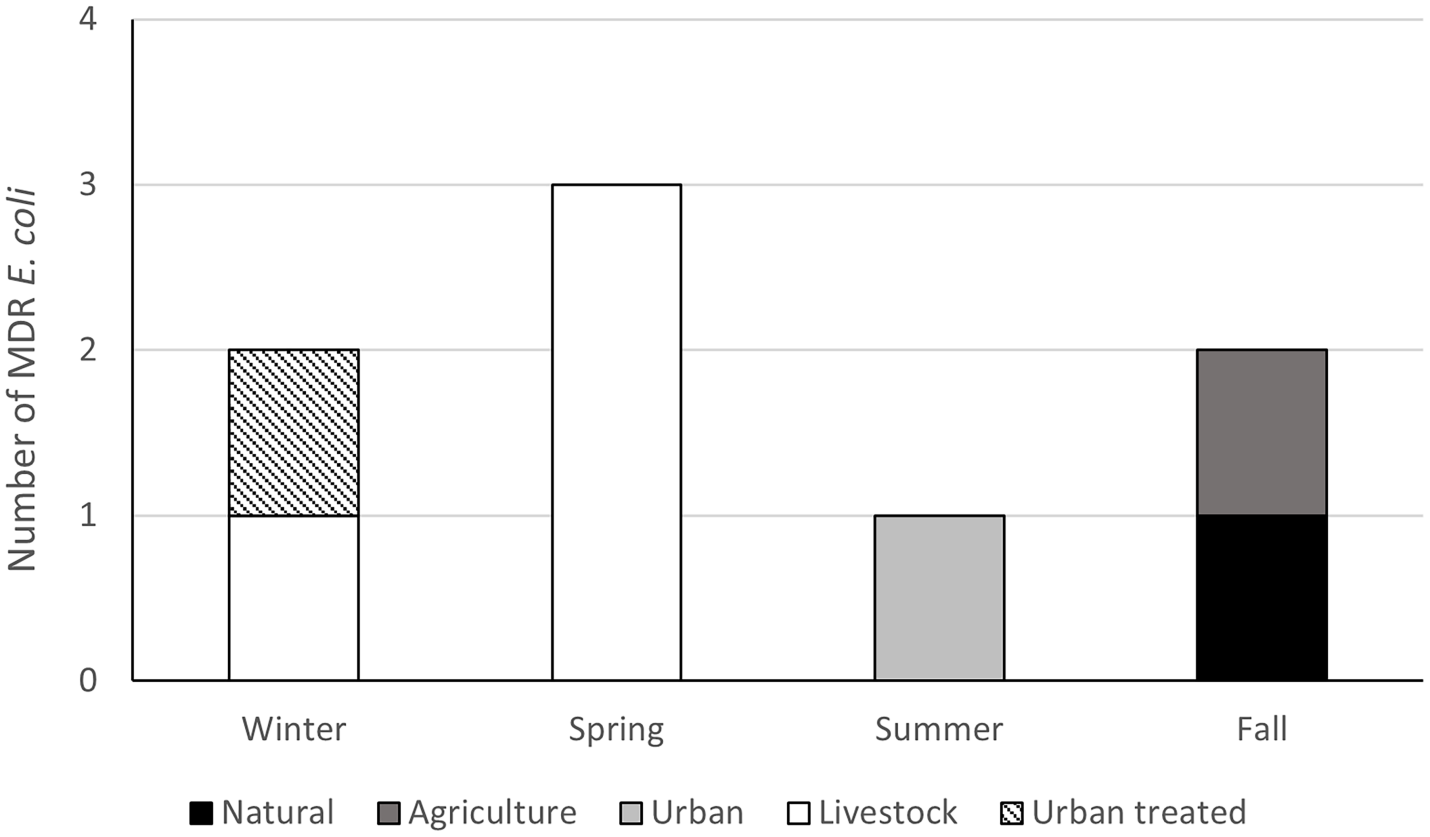
Number multi-drug resistant (MDR) *Escherichia coli* by land use and season. Isolates resistant to three or more antimicrobial classes were cataloged as MDR following previously standardized criteria (Magiorakos et al., 2012).

**Figure 7:**
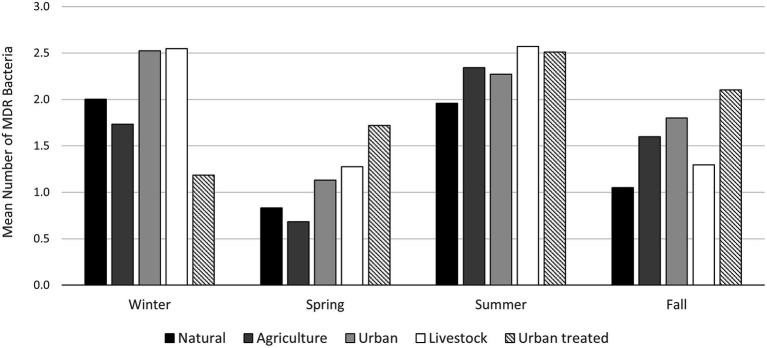
Mean number of environmental MDR bacteria by land use and season. Isolates resistant to three or more antimicrobial classes were cataloged as MDR following previously standardized criteria (Magiorakos et al., 2012).

To evaluate antimicrobial resistance in enteric bacteria, *E. coli* were isolated, and their resistance profile was characterized. Twenty-eight out of 48 samples analyzed (58.3%) contained Gram-negative bacteria resistant to at least one of the antibiotics tested (Ciprofloxacin, Cefotaxime, and Tetracycline). We obtained 66 isolates; 26 (lactose positives) isolates were characterized at the species level by MALDI-TOF MS. Finally, we identified eight *E. coli* isolates and three of them showed a multi-drug resistant profile (Figure 6; Supplementary Table S4). Regarding the environmental bacteria, 2,631 morphotypes were isolates at different temperatures (37°C, 25°C, and 15°C) and media. The major number of colonies were obtained at 25°C and in the R2A medium, demonstrating that the optimal temperature for environmental bacterial growth is 25°C. To determine the AMR profile, the MICs for Ampicillin (AMP), Erythromycin (ERY), Ceftazidime (CAZ), Imipenem (IMI), Ciprofloxacin (CIP), Gentamicin (GEN), and Tetracycline (TET) were determined. Bacteria that showed resistance to at least three antibiotics of different families were classified as MDR bacteria. The percentage of MDR-bacteria was evaluated by season and land use and summer was the season with higher values in most of the land use analyzed (>2.5%), followed by the winter season where a high percentage of resistance was observed in both urban and livestock land uses (>2%; Figure 7).

## Discussion

The contamination of water with fecal material represents a problem for public health. Determining the source of fecal material could be useful to reduce the load of fecal material in rivers to designing mitigation measures that provide water with adequate quality for agriculture and livestock. In this study, fecal contamination levels were determined, and the probable sources of contamination were identified through an MST analysis, together with antimicrobial resistance, in two main rivers of central Chile based on land use. The main finding of our study suggests that: (i) microbiological water quality is affected by environmental conditions, particularly land use and season, and (ii) identification of *Cryptosporidium* and *Giardia* species provides information about the possible host source of the fecal contamination. Microbial water quality in the Maipo River was worse than in the Maule River; this was expected due to the larger human population in the Maipo (approx. 9 million) compared to the Maule River (approx. 1 million) basin (INE, Instituto Nacional de Estadísticas, 2017). Indeed, past studies have linked impaired water quality to increased anthropogenic disturbance (Lenart-Boroń et al., 2017; Cui et al., 2019; Yuan et al., 2019). For example, Yuan et al. (2019) examined water quality in canals in Suzhou, China, and found that greater urbanization was associated with higher microbial loads, including fecal markers and human pathogens. Similarly, Cui et al. (2019) examined 41 water samples of 15 rivers urban rivers located in Changzhou City, China and reported that the samples harbored diverse enteric pathogens, which were highly correlated to the human fecal marker abundances. Correspondingly, Lenart-Boroń et al. (2017) evaluated the water quality at 21 sites in major rivers of the Podhale region (southern Poland) for a year. Their results suggest that an increased share of built-up areas and arable land results in significant deterioration of water quality and concluded that point sources of pollution (e.g., effluents from treatment plants or discharge of untreated sewage from households) were more important than non-point sources (e.g., surface runoff). These findings are supported by the results of the conditional trees in the present study; specifically, high fecal coliform levels and a greater likelihood of detecting *Giardia* were observed for samples collected from sites with anthropogenic influence (e.g., urban and agricultural). Based on our findings efforts to mitigate fecal contamination in Central Chile should focus on the Maipo River, and specifically on sources of contamination in urban and agricultural regions. Further studies are needed to identify specific sources of contamination in urban and agricultural reaches of the Maipo River.

The correlation between fecal coliforms and parasites was evaluated. While we found evidence of a significant correlation between fecal coliform and *Giardia* levels, there was correlation between fecal coliform and *Cryptosporidium* levels, which may be driven by the small number of *Cryptosporidium*-positive samples in the present study. Indeed, a review that assessed relationships between FIBs and pathogens in water hypothesized that the failure to find associations between FIBs and the target was due to insufficient data (Wu et al., 2011). However, it is also important to recognize, that failure to find evidence of a relationship may also be due to the absence of a relationship. Indeed, several recent studies have shown that pathogen-FIB relationships are dependent on the waterway and/or environmental context of the sampling site and sampling event (McEgan et al., 2013; Weller et al., 2020b). Overall, our finding of an association between FIB levels and *Giardia* levels is consistent with past studies. Many were analyzed in review studies on the relationship between FIBs and pathogens and parasites in recreational water (Korajkic et al., 2018). Specifically, this review found that almost 50% of the 73 studies considered performed statistical analysis to determine if there was a significant association between FIB and other pathogens (including parasites). Only considering freshwater studies (*n* = 41), a positive association with pathogens was reported in two-thirds of them (*n* = 23), and the remaining studies did not support statistical analysis (Korajkic et al., 2018).

*Cryptosporidium* spp. and *Giardia* spp. have been extensively used to perform MST analysis (Prystajecky et al., 2014; dela Peña et al., 2021; González-Fernández et al., 2021). During this study, *Cryptosporidium* spp. and *G. duodenalis* were determined in water samples. Most *Cryptosporidium* spp. and *G. duodenalis* assemblages corresponded to subtypes or species that are not host-specific, such as *C. parvum* and *G. duodenalis* assemblages A and B, respectively (Xiao et al., 2000, 2004; Cacciò et al., 2005; Monis et al., 2009). Even though the amplicon used in our study generated sequences too short to allow differentiation between assemblages A or B, it does not affect our result interpretation as both assemblages are not host-specific. Furthermore, despite that *G. duodenalis* A and B are host-unspecific, it has been reported that humans and livestock are among the main hosts affected by these assemblages (Cacciò et al., 2005; Monis et al., 2009). Nevertheless, it was possible to identify a *C. canis* (in one sample from natural land use), *C. meleagridis* (in one sample from agricultural land use), and *C. andersoni* (in two samples from urban land use), which are specific to dogs, humans, and birds, and cattle, respectively (Xiao et al., 2000, 2004; Cacciò et al., 2005). *Cryptosporidum canis* has been reported to infect wild canines such as foxes (Zhang et al., 2016; Papini and Verin, 2019). Since various canines such as foxes (*Lycalopex culpaeus*) and free-ranging dogs, among others, inhabit the natural sections of the river systems studied (Cevidanes et al., 2020; Benavides et al., 2021), this may explain the detection of *C. canis* in the present study from samples collected from sites where the predominant land use is natural. *G. duodenalis* (assemblage G) associated with rats and/or mice was detected in the livestock/forestry area in the Maule River (Cacciò et al., 2005; Monis et al., 2009). Therefore, based on our results, *Cryptosporidum* spp. and *G. duodenalis* provided information about the possible host source of fecal contamination in rivers. To our knowledge, a standard profile for *Cryptosporidum* spp. does not exist. Therefore, we cannot make comparisons. Our study had a low likelihood of detecting *Cryptosporidium*, with many samples presenting non-detection of this parasite. A possible explanation of this result is that metallic sediments present in the river water samples may negatively affect the performance of the IMS technique and, therefore, hamper the recovery of *Cryptosporidium spp*. Therefore, improvements must be conducted in the detection protocol as we presented complications when using the immunomagnetic separation technique (IMS) as the rivers in Chile naturally have high concentrations of metallic sediments (copper, among others; Ghorbani and Kuan, 2016), which might have interfered with the detection of these protozoa, causing a possible underestimation in the detection. This could explain the low levels and identification of *Cryptosporidum* spp. in river water samples in our studies. Further studies should be conducted to evaluate whether the metallic sediments of mining activities interfere with detection methods involving magnetics beads and how to overcome this problem.

In past studies, *Cryptosporidum* oocysts and *Giardia* cysts have been detected in treated effluents of WWTP (Kitajima et al., 2014; Domenech et al., 2018), and reports indicate that some treatments along the WWTP process (stabilization ponds, constructed wetland, ultrafiltration, and UV irrigation) are found to be effective for the removal of *Cryptosporidium* oocysts, while other treatments (activates sludge, high-rate filtration, and chlorine disinfection) are ineffective (Nasser, 2016). In Chile, the removal of protozoa from effluent and the use of UV light, ozone, or other treatments besides chlorine in the tertiary treatment is not mandatory for WWTP (Ministerio Secretaría General de la Presidencia, 2001); therefore, it is highly likely that protozoa are not completely removed from the treated effluent. We collected river water samples upstream and downstream of the discharge of treated effluent of WWTP to determine the effect on the number and species of protozoa released in the river water. It is worth mentioning that once the effluent is released from the treatment plant by an underground pipe, it flows through an open canal for approximately 30 m before reaching the river. Along the trajectory of the effluent, it crosses some households that have horses, chickens and/or dogs. Therefore, fecal or protozoan contamination may come from flowing through this canal rather than from the WWTP. This could explain the presence of *G. duodenalis* assemblages A or B without a specific host that is probably from human sources. Considering this finding, we suggest that the effluents from the WWTP should be discharged directly into the rivers through underground pipes rather than into open canals to evaluate whether these protozoa are coming from the effluent of wastewater treatment, therefore associated with humans, or are coming from other animal sources (Encyclopedia Britannica, 2021).

Aquatic ecosystems are considered the most important matrices for the release, mixing, persistence, and dissemination of ARB (Baquero et al., 2008; Taylor et al., 2011; Yang et al., 2018); especially freshwater bodies since mixing occurs with a high risk of genetic exchange between coliforms and environmental bacteria (Kraemer et al., 2019; Nnadozie and Odume, 2019). Determining the impact of the mixture between environmental bacteria and bacteria that arise from contamination by human activity, like coliforms, is one of the great challenges in the AMR phenomenon. In this study, we have shown that the greatest amount of environmental ARB and coliforms are isolated in the summer season, which could increase the probability of genetic exchange between these two microbiomes. The same effect could be observed when the variable land use is analyzed. In both cases, soils classified as urban presented the highest levels of fecal coliforms and environmental ARB, which further enables genetic exchange between these two bacterial groups. Understanding the factors that favor the mobilization of antibiotic resistance determinants between different microbiomes is pivotal in the resistance phenomenon. In this study, we have determined that the summer season and the type of urban land use are the factors where the greatest levels of coliforms were counted and where the largest number of environmental ARBs were isolated; this could favor the genetic exchange between these two microbiomes, generating a major problem in the AMR phenomenon.

## Conclusion

Our results indicate that river water used as irrigation water has high levels of fecal coliforms, with many sites evaluated having noncompliance with the Chilean water quality standard, protozoa, and AMR. Fecal contamination (level or based on the compliance of a water quality regulation), protozoa levels, and ARB presence in river water depended on the interaction of environmental factors such as river, land use, and season. In our study, the Maipo River had higher levels of coliforms (and noncompliance with the Chilean water quality criteria), protozoa detection, and ARB than the Maule River. The land use that contributed most to the fecal and protozoa contamination of river water was after the discharge of wastewater treatment plants (urban treated), urban land use, and agriculture. Therefore, the environmental factors of land use, river, and season should be considered when designing studies to evaluate water contamination as the microbial contamination varies according to the human activity in the area of interest.

The presence of different species of *Cryptosporidium* spp. and assemblages of *Giardia duodenalis* indicate that humans, cattle, birds, rats/mice, and dogs are the most probable source of origin of the fecal contamination. Furthermore, these protozoa are known to be zoonotic and therefore may pose a potential public health threat if this water is directly or indirectly consumed. Therefore, based on our results, we can conclude that *Cryptosporidium* spp. and *G. duodenalis* detection and land use can be used as MST tools to provide information on the possible source of fecal contamination. Finally, we can say that the highest levels of fecal coliforms were detected in both the summer season and the land use classified as urban, and the largest amount of environmental ARB was isolated, increasing the probabilities of mobilization and dispersion of genetic elements involved in AMR. These data could aid in implementing measures to mitigate the dispersion of coliforms and ARB, thus reducing risks to public health.

## Data Availability Statement

The datasets presented in this study can be found in online repositories. The names of the repository/repositories and accession number(s) can be found in the article/Supplementary Material.

## Author Contributions

CD-G, CB, LV, MK, AM-S, JO-P, and ADA designed the study, planned the field work logistics, and wrote the manuscript. DW and AA ran the statistical analysis, while CD-G constructed the phylogenetic trees. CD-G, CB, MS-C, EE, AA, MK, and ADA conducted experiments. DW, MS-C, EE, WS, MK, AM-S, JO-P, and ADA designed experiments and critically reviewed the manuscript. All authors contributed to the article and approved the submitted version.

## Funding

We acknowledge the financial support received from the FONDECYT 11160116 and 1221536 to ADA, FONDECYT 1181167 to AM-S, and ANID Millennium Science Initiative/Millennium Initiative for Collaborative Research on Bacterial Resistance, MICROB-R, NCN17_081 to ADA, AM-S, and JO-P. Data analysis was supported by the National Institute of Environmental Health Sciences of the National Institutes of Health (NIH) under award number T32Es007271.

## Conflict of Interest

The authors declare that the research was conducted in the absence of any commercial or financial relationships that could be construed as a potential conflict of interest.

## Publisher’s Note

All claims expressed in this article are solely those of the authors and do not necessarily represent those of their affiliated organizations, or those of the publisher, the editors and the reviewers. Any product that may be evaluated in this article, or claim that may be made by its manufacturer, is not guaranteed or endorsed by the publisher.

## Supplementary Material

The Supplementary Material for this article can be found online at: https://www.frontiersin.org/articles/10.3389/fmicb. 2022.768527/full#supplementary-material
