# Supplementary material for: Humans and Hoofed Livestock Are the Main Sources of Fecal Contamination of Rivers Used for Crop Irrigation: A Microbial Source Tracking Approach
Source: Front Microbiol. 2022 Jun 30;13:768527. doi: 10.3389/fmicb.2022.768527 (PMC9279616; doi:10.3389/fmicb.2022.768527)
Supplement: Supplementary file 1 [file Data_Sheet_1.DOCX]

**SUPLEMENTARY FIGURES**

ARTICLE TITLE: **Human and hoofed livestock are the main sources of fecal contamination of rivers used for crop irrigation: A microbial source tracking approach**

Constanza Díaz-Gavidia^1,6^, Carla Barría^1,6^, Daniel Weller^2^, Marilia Salgado^1^, Erika M. Estrada^3^, Aníbal Araya^4,6^, Leonardo Vera^7^, Woutrina Smith^8^, Minji Kim^9^, Andrea I. Moreno-Switt^6,10^, Jorge Olivares-Pacheco^4,6,^ Aiko D. Adell^1,6*^

^1^ Escuela de Medicina Veterinaria, Facultad de Ciencias de la Vida, Universidad Andres Bello, Santiago, Chile

^2^Department of Environmental and Forest Biology, State University of New York, College of Environmental Science and Forestry, Syracuse, NY USA

^3^ Department of Food Science and Technology, Virginia Tech, Painter, VA 23420, USA

^4^ Grupo de Resistencia Antimicrobiana en Bacterias Patógenas y Ambientales (GRABPA), Instituto de Biología, Pontificia Universidad Católica de Valparaíso, Chile.

^5^ Facultad de Medicina, Universidad del Desarrollo, Chile

^6^ Millennium Initiative for Collaborative Research on Bacterial Resistance (MICROB-R), Santiago, Chile

^7^ Escuela Ingeniería Ambiental, Facultad de Ciencias de la Vida, Universidad Andres Bello, Santiago, Chile

^8^ One Health Institute, School of Veterinary Medicine, One Shields Avenue, University of California, Davis, CA 95616, USA

^9^ Department of Civil and Environmental Engineering, University of California, Davis, One Shields Avenue, Davis, CA 95616, USA.

^10^ Escuela de Medicina Veterinaria, Facultad de Agronomía e Ingeniería Forestal, Facultad de Ciencias Biológicas y Facultad de Medicina, Pontificia Universidad Católica de Chile, Santiago, Chile

Supplementary Table S1: Nucleotide sequences of PCR primers used to identify *Cryptosporidium* and *Giardia* species in river water samples.

| **Amplification target** | **Target Gene** | **Primer** | **Primer sequence (5′–3′)** | **PCR Final Product length (bp)** |
| --- | --- | --- | --- | --- |
| *Cryptosporidium*spp*.* | Morgan 18S rRNA^1^ | Forward | AGTGACAAGAAATAACAATACAGG | 298-bp |
|  |  | Reverse | CCTGCTTTAAGCACTCTAATTTTC |  |
|  | Xiao 18S rRNA^2,3^ | External Forward^2^ | TTCTAGAGCTAATACATGCG | 826-864-bp |
|  |  | External Reverse^3^ | CCCTAATCCTTCGAAACAGGA |  |
|  |  | Internal Forward^2^ | GGAAGGGTTGTATTTATTAGATAAAG |  |
|  |  | Internal Reverse^2^ | AAGGAGTAAGGAACAACCTCCA |  |
| *Giardia*spp*.* | 16S-ssuRNA^4^ | External Forward | AAGTGTGGTGCAGACGGACTC | 292-bp |
|  |  | External Reverse | CTGCTGCCGTCCTTGGATGT |  |
|  |  | Internal Forward | CATCCGGTCGATCCTGCC |  |
|  |  | Internal Reverse | AGTCGAACCCTGATTCTCCGCCAGG |  |
|  | GDH^5^ | External Forward | TCAACGTYAAYCGYGGYTTCCGT | 432-bp |
|  |  | Internal Forward | CAGTACAACTCYGCTCTCGG |  |
|  |  | Internal Reverse | GTTRTCCTTGCACATCTCC |  |

^1^ Morgan et al., 1997

^2^ Xiao et al 1999

^3^ Xiao et al 2000

^4^ Appelbee et al., 2003

^5^ Read et al., 2004

Supplementary table S2: Accession numbers for *Cryptosporidium* and *Giardia* isolates found in this study.

| Accession Number | Organism |
| --- | --- |
| MT476873 | *Cryptosporidium andersoni* |
| MT476874 | *Cryptosporidium canis* |
| MT476875 | *Cryptosporidium meleagridis* |
| MT476876 | *Cryptosporidium andersoni* |
| MT476877 | *Cryptosporidium parvum* |
| MT484072 | *Giardia duodenalis* assemblage A or B |
| MT484073 | *Giardia duodenalis* assemblage A or B |
| MT484074 | *Giardia duodenalis* assemblage A or B |
| MT484075 | *Giardia duodenalis* assemblage G |
| MT484076 | *Giardia duodenalis* assemblage A or B |
| MT484077 | *Giardia duodenalis* assemblage A or B |
| MT484078 | *Giardia duodenalis* assemblage A or B |
| MT484079 | *Giardia duodenalis* assemblage A or B |
| MT484080 | *Giardia duodenalis* assemblage A or B |
| MT484081 | *Giardia duodenalis* assemblage A or B |
| MT484082 | *Giardia duodenalis* assemblage E |
| MT484083 | *Giardia duodenalis* assemblage B-III/B-IV |
| MT484084 | *Giardia duodenalis* assemblage A or B |
| MT484085 | *Giardia duodenalis* assemblage A or B |
| MT484086 | *Giardia duodenalis* assemblage E |
| MT484087 | *Giardia duodenalis* assemblage A or B |
| MT484088 | *Giardia duodenalis* assemblage A or B |
| MZ546934 | *Giardia duodenalis* assemblage B-IV |
| MZ546935 | *Giardia duodenalis* assemblage A-II |
| MZ546936 | *Giardia duodenalis* assemblage A-II |
| MZ546937 | *Giardia duodenalis* assemblage B-IV |

Supplementary table S3: *Cryptosporidium* species and *Giardia duodenalis* assemblages identified by PCR sequencing in each river, land use and season during the 2-years study

| River | Land Use | Sampling Period | | | | | | | |
| --- | --- | --- | --- | --- | --- | --- | --- | --- | --- |
|  |  | Winter 2017 | Spring 2017 | Summer2018 | Fall 2018 | Winter 2018 | Spring 2018 | Summer 2019 | Fall 2019 |
| Maule | Natural | n.d. | n.d. | n.d. | n.d. | n.d. | n.d. | n.d. | n.d. |
|  | Agricultural | n.d. | n.d. | n.d. | n.d. | n.d. | n.d. | n.d. | n.d. |
|  | Urban | n.d. | ***Gd* – A/B^3^** | n.d. | n.d. | n.d. | n.d. | n.d. | n.d. |
|  | Livestock/Forestry | n.d. | n.d. | n.d. | n.d. | n.d. | n.d. | n.d. | ***Gd* – G^3^** |
| Maipo | Natural | n.d. | n.d. | n.d. | n.d. | n.d. | ***Gd* – A/B^3^** | n.d. | n.d. |
|  | Treated urban | ***Gd* – A/B^3^** | n.d. | ***Gd* – A/B ^3^** | ***Gd* – A/B^3^**  ***Gd-BIV*^4^** | ***Gd* – A/B^3^** | ***Gd* – A/B^3^** | n.d. | ***Gd* – A/B^3^** |
|  | Natural | n.d. | n.d. | n.d. | n.d. | n.d. | n.d. | n.d. | ***C. canis*^5^** |
|  | Agricultural | n.d. | n.d. | n.d. | n.d. | n.d. | n.d. | n.d. | n.d. |
|  | Treated urban | n.d. | ***Gd* – A/B^3^** | ***Gd* – A/B^3^** | n.d. | n.d. | n.d. | n.d. | n.d. |
|  | Agricultural | n.d. | ***Gd* – A/B^3^** | n.d. | n.d. | n.d. | ***Gd* – A/B^3^** | ***Gd* – A/B^3^** | ***C.*** ***meleagridis*^5^** |
|  | Urban | ***C. andersoni* ^5^**  ***Gd* – A/B^3^**  ***Gd* – BIV^4^** | ***Gd* – A/B^3,^**  ***Gd* – AII^4^** | n.d. | n.d. | n.d. | n.d. | n.d. | ***C. andersoni* ^5^** |
|  | Livestock | n.d. | ***Gd* – A/B^3^**  ***Gd* – AII^4^** | n.d. | n.d. | n.d. | n.d. | n.d. | ***C. parvum*^5^** |

*^1^Gd:* corresponds to *Giardia duodenalis,* while the letters A, A or B (A/B), BIII, BIV, E and G correspond to the genetic groups or assemblages

^2^ n.d.: Not detected.

^3^ Positive for ssurRNA – *Giardia*

^4^ Positive for GDH – *Giardia*

^5^ Positive for Xiao et al 1999 and 2000


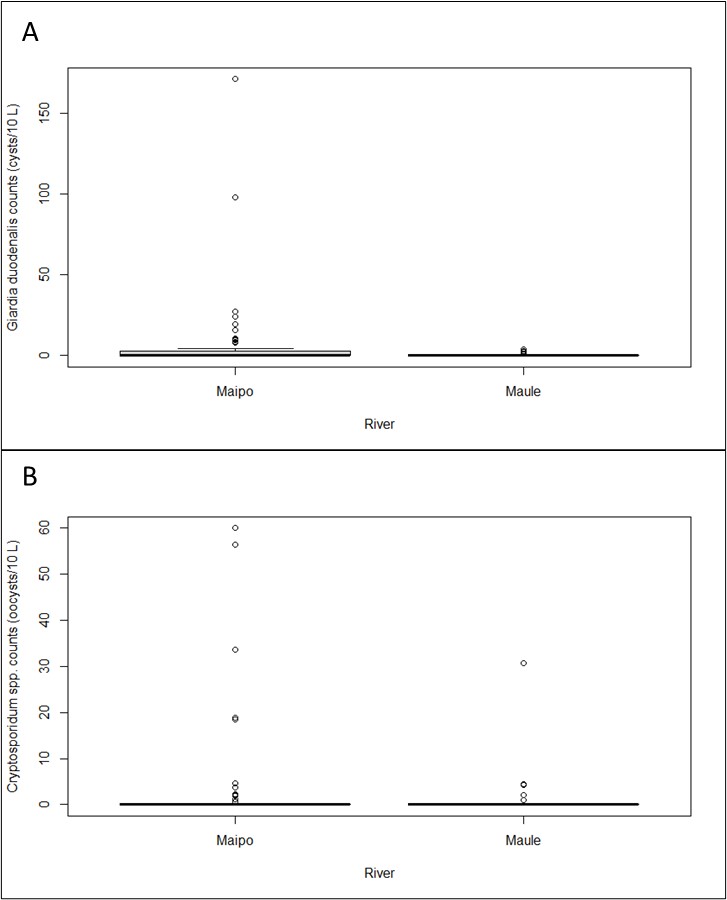
Supplementary Figure S1: Mean number of protozoa [(oo)cysts/ 10L)] from water by river. (A) *Giardia duodenalis* levels (cysts/10 L) in water samples and (B) *Cryptosporidum* spp. levels (oocysts/10 L) in water samples. The box plots were created using RStudio v1.1.456 open-source software (RStudio team, 2020)

Supplementary Table S4: Antimicrobial resistant profile of *E. coli* isolates from water^1,2^

|  | AMP | CFZ | GEN | AMK | AMC | CXM | FEP | FOX | CRO | CIP | IMP | SXT | ATM | CHL | TET |
| --- | --- | --- | --- | --- | --- | --- | --- | --- | --- | --- | --- | --- | --- | --- | --- |
| *E. coli* ^3^ | **R** | **R** | S | S | I | **R** | **R** | S | **R** | S | S | S | **R** | S | S |
| *E. coli* ^3^ | **R** | **R** | S | S | **R** | **R** | **R** | **R** | **R** | S | I | S | **R** | S | S |
| *E. coli* ^3^ | **R** | **R** | S | S | **R** | **R** | **R** | **R** | **R** | S | I | S | **R** | S | S |
| *E. coli* | S | S | S | S | S | S | S | S | S | S | S | S | S | S | **R** |
| *E. coli* | S | S | S | S | S | S | S | S | S | S | S | S | S | S | **R** |
| *E. coli* ^3^ | **R** | S | **R** | S | S | S | S | S | S | **R** | S | **R** | S | S | **R** |
| *E. coli* ^3^ | **R** | I | **R** | S | S | S | S | S | S | **R** | S | **R** | S | S | **R** |
| *E. coli* ^3^ | **R** | I | **R** | S | S | S | S | S | S | **R** | S | **R** | S | S | **R** |

^1^ The presence of *E. coli* was evaluated only during the first year of sampling

^2^ The letters in the table correspond to the level of AMR: R = resistant, I = intermediate resistance, and S = sensible

^3^ Multi-drug resistant isolates

AMP = Ampicillin; CFZ = Cefazolin; GEN = Gentamicin; AMK = Amikacin; AMC = Amoxicillin-Clavulanate; CXM = Cefuroxime; FEP = Cefepime; FOX = Fosfomycin/Trometamol; CRO = Ceftriaxone; CIP = Ciprofloxacin; IMP = Imipenem; SXT = Trimethoprim/sulfamethoxazole; ATM = Aztreonam; CHL = Chloramphenicol; TET= Tetracycline
